# Combining RNA Interference and RIG-I Activation to Inhibit Hepatitis E Virus Replication

**DOI:** 10.3390/v16091378

**Published:** 2024-08-29

**Authors:** Mathias Ziersch, Dominik Harms, Lena Neumair, Anke Kurreck, Reimar Johne, C.-Thomas Bock, Jens Kurreck

**Affiliations:** 1 Applied Biochemistry, Institute of Biotechnology, Technische Universität Berlin, 13355 Berlin, Germany; mathias.ziersch@tu-berlin.de (M.Z.); lena.neumair@chem.tu-berlin.de (L.N.); 2 Department of Infectious Diseases, Division of Viral Gastroenteritis and Hepatitis Pathogens and Enterovirus, Robert Koch Institute, 13353 Berlin, Germany; harmsd@rki.de (D.H.); bockc@rki.de (C.-T.B.); 3 Bioprocess Engineering, Institute of Biotechnology, Technische Universität Berlin, 13355 Berlin, Germany; anke.wagner@tu-berlin.de; 4 BioNukleo GmbH, Ackerstrasse 76, 13355 Berlin, Germany; 5 Department of Biological Safety, German Federal Institute for Risk Assessment, 12277 Berlin, Germany; reimar.johne@bfr.bund.de

**Keywords:** HEV, siRNA, RNAi therapy, RIG-I, RNA 5′triphosphate

## Abstract

Hepatitis E virus (HEV) poses a significant global health threat, with an estimated 20 million infections occurring annually. Despite being a self-limiting illness, in most cases, HEV infection can lead to severe outcomes, particularly in pregnant women and individuals with pre-existing liver disease. In the absence of specific antiviral treatments, the exploration of RNAi interference (RNAi) as a targeted strategy provides valuable insights for urgently needed therapeutic interventions against Hepatitis E. We designed small interfering RNAs (siRNAs) against HEV, which target the helicase domain and the open reading frame 3 (ORF3). These target regions will reduce the risk of viral escape through mutations, as they belong to the most conserved regions in the HEV genome. The siRNAs targeting the ORF3 efficiently inhibited viral replication in A549 cells after HEV infection. Importantly, the siRNA was also highly effective at inhibiting HEV in the persistently infected A549 cell line, which provides a suitable model for chronic infection in patients. Furthermore, we showed that a 5′ triphosphate modification on the siRNA sense strand activates the RIG-I receptor, a cytoplasmic pattern recognition receptor that recognizes viral RNA. Upon activation, RIG-I triggers a signaling cascade, effectively suppressing HEV replication. This dual-action strategy, combining the activation of the adaptive immune response and the inherent RNAi pathway, inhibits HEV replication successfully and may lead to the development of new therapies.

## 1. Introduction

Hepatitis E virus (HEV) is a significant contributor to acute viral hepatitis cases globally, affecting approximately 20.1 million individuals annually. It is estimated that HEV infection causes 70,000 deaths due to liver failure and 3000 miscarriages each year [1]. Various HEV strains with different regional distributions have been isolated. The prevalence of genotype 1 (HEV-1) is notably high in Africa and Asia, while genotype 2 (HEV-2) predominates in specific African regions and Mexico. The most common mode of transmission is through contaminated drinking water, which perpetuates outbreaks through the fecal–oral route [2]. HEV-3 and HEV-4 are primarily observed in developed countries. HEV-4 is geographically restricted to Southeast Asia, while HEV-3 is distributed worldwide, and both are transmitted zoonotically via food-borne routes [3,4]. A recent meta-analysis indicates that approximately 12.5% of the global population experiences HEV infection during their lifetimes, as discerned through positive anti-HEV IgG antibody tests [5].

The majority of HEV infections resolve spontaneously. However, 5–30% may progress to acute icteric hepatitis, usually within six weeks in immunocompetent individuals [6]. Chronic hepatitis may ensue among certain immunocompromised cohorts, notably solid and hematopoietic transplant recipients, in human immunodeficiency virus (HIV) co-infections or during chemotherapy [7,8,9]. Chronic hepatitis E is defined by the persistence of HEV RNA in the blood or stool for a period exceeding three months, with the potential for dire complications, including liver cirrhosis or failure, if viremia persists [10,11]. Severe acute HEV-1 infections are particularly prevalent among pregnant women in their third trimester, with the potential for significant complications, including liver failure, fetal loss, and mortality rates of up to 25% [12].

**Figure 1:**
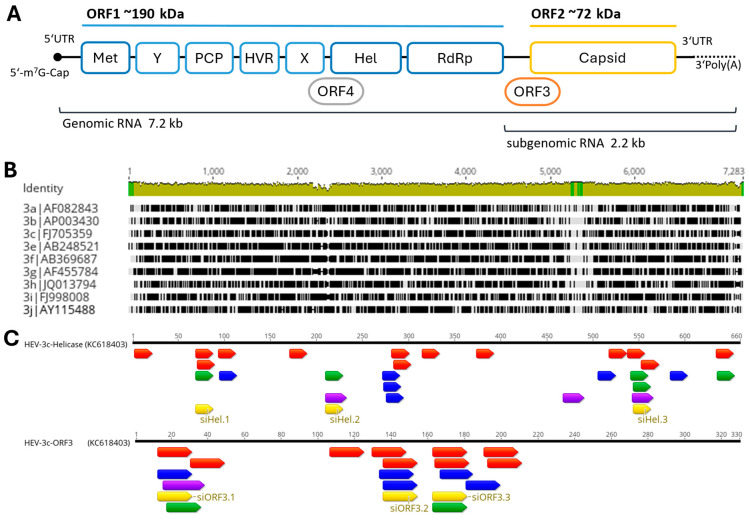
Organization of the HEV genome and siRNA target selection. (**A**) Overview of the HEV genome. The (+)ssRNA genome is 7.2 kb long. It has a 7-methylguanosine cap at the 5′ end and polyadenylation at the 3′-end. There are three conserved ORFs. ORF1 encodes the non-structural polyproteins and has several functional domains: methyltransferase (Met), Y domain, papain-like cysteine protease (PCP), hypervariable region (HVR), X domain, helicase (Hel), and RNA-dependent RNA polymerase (RdRP). ORF2 encodes the capsid structural protein. ORF3 encodes a multi-functional phosphoprotein. Expression is mediated by a 7.2 kb subgenomic bicistronic RNA. In addition to these three ORFs, HEV-1 has an ORF4 that overlaps the X and Hel domains. (**B**) Alignment of all HEV-3 subgenotypes to identify conserved regions within the genotype. Non-matching sequences are shown in black—conserved regions in gray. (**C**) Representation of suitable siRNA target sequences in the helicase and ORF3 regions. Identification was performed using the online programs Eurofins Genomics (blue), Horizon Discovery (red), OligoWalk Web Server (green), and siDirect2.0 (purple). Selected siRNA target sequences are shown in yellow.

HEV is an icosahedral RNA virus excreted as non-enveloped particles (27–34 nm) in stool via the bile. Quasi-enveloped particles are found in the blood. Its positive-sense (+) single-stranded (ss) RNA genome (7.2 kb) contains a 5′-7-methylguanosine (5′-m7G)-cap, followed by a 5′-untranslated region (UTR), three open reading frames (ORFs), and polyadenylation (Figure 1A). ORF1 encodes non-structural polyproteins, including functional domains like the methyltransferase (Met), X domain, helicase (Hel), and RNA-dependent RNA polymerase (RdRP). A cis-acting element between ORF1 and ORF2 controls subgenomic RNA expression [13]. This RNA codes for ORF2 (capsid protein) and ORF3 (relevant for virus egress) [14,15,16]. Mutations that impair the activity of ORF3 reduce the release of virions. In fact, a mutated HEV without ORF3 was unable to cause a productive infection in mice [17,18]. HEV-1 putative ORF4, regulated by an internal ribosome entry site (IRES), overlaps with X and Hel domains and is activated under endoplasmic reticulum (ER) stress [19].

There are only limited options for the treatment of HEV infections. Reduction of immunosuppressive drugs in patients with HEV-induced chronic hepatitis post-organ transplantation clears the virus in over 30% of cases [20]. Pegylated interferon α (PegIFNα) serves as an alternative, but it is not recommended in kidney transplant patients due to an increased risk of acute renal failure [21,22]. Currently, there are no approved drugs for the treatment of HEV, but ribavirin (RBV), a guanosine nucleoside analog, is sometimes used off-label for chronic hepatitis E treatment [23,24]. However, RBV therapy, typically lasting three to six months, can induce dose-dependent anemia and other adverse effects [25]. There are also alarming indications that RBV treatment may favor HEV mutagenesis, potentially reducing treatment efficacy and the emergence of escape mutants [26,27,28].

A systemic review by Gorris et al. showed that chronic HEV infections were predominantly of the HEV-3 genotype (97%). Negative serum HEV RNA levels for a minimum of 3 months post-RBV-treatment cessation were achieved in about 76% of patients after RBV treatment. A relapse was experienced in 18%, with further treatment needed, and a non-response was observed in 6% of all cases [29]. Patients failing ribavirin therapy have no further treatment options.

One effective strategy to hinder viral replication involves employing RNA interference (RNAi), a well-conserved and highly efficient mechanism of post-transcriptional gene silencing. RNAi is initiated by short double-stranded RNA molecules, such as small interfering RNA (siRNA), which prompt the degradation of mRNA in a sequence-specific manner, ultimately leading to the specific inhibition of gene expression [30,31].

Several studies have explored RNAi strategies against the HEV. Huang et al. targeted the RdRP sequence in HEV-4 [32], while Kumar et al. aimed at the sequence in HEV-1 [33]. Huang et al. targeted ORF2 in HEV-4 [34], and Liu et al. focused on ORF3 in HEV-4 [35]. Zhang et al., utilizing adeno-associated virus (AAV), investigated multiple HEV sequences (except ORF3) in HEV-3 [36]. A summary can be seen in Table 1.

In our study, we focused on conserved regions in the HEV-3 genome, as the previously mentioned RBV treatment may lead to mutations that can alter the target sequence of siRNAs, leading to a significant decrease in RNAi efficacy [37,38].

The cytosolic pattern recognition receptor RIG-I is essential for cellular defense against RNA viruses by initiating the early innate immune response, including the activation of type-I-interferon. It detects double- and single-stranded RNA with a 5′-triphosphate group, which acts as a ligand for RIG-I [39,40,41]. Findings demonstrate the negative impact of HEV proteins on the RIG-I signaling pathway [42,43,44], suggesting that HEV may circumvent the host antiviral response via this pathway. Xu et al. used lentiviral transduction to overexpress RIG-I in HEV-infected Huh7.5, A549, and HepaRG cells and significantly reduced HEV replication. This is corroborated by the observation that targeted RNAi-mediated silencing of RIG-I in A549 cells led to a significant increase in HEV RNA levels. Moreover, they reported that the activation of RIG-I signaling by 5′-triphosphate RNA diminished HEV replication in infected A549 cells [45]. Inhibition of influenza A virus [46] and hepatitis B virus replication [47,48] was observed when employing a 5′-triphosphate-modified siRNA, as opposed to a standard siRNA. However, no such strategy has yet been reported for HEV.

The present study describes the design of an efficient siRNA against a conserved region of the HEV genome. In an initial experiment, we show that this siRNA prevents viral spread when transfected into the cells prior to infection. Importantly, it also efficiently inhibits the virus in a persistently infected cell line. The persistent infection represents the chronic infection found in patients. To prevent viral escape by simple mutations in the siRNA target site, we produced a bivalent therapy against HEV by combining an siRNA with a triphosphate at the 5′ end of the sense strand. The triphosphate inhibited the virus, even when attached to an siRNA control sequence. We thus developed a highly efficient silencing strategy against HEV, which is based on two mechanisms to minimize the risk of viral escape.

## 2. Materials and Methods

### 2.1. Design of siRNAs and Plasmid Construction

All HEV-3 subtype reference genomes (Table S1) were aligned using the Geneious Prime 2021.2.2 (GraphPad Software LLC, Boston, MA, USA) to identify conserved regions [49]. The sequence of the helicase in ORF1 and the sequence for the multi-functional ORF3 protein on the HEV genome were identified as suitable targets for the siRNAs. The patient-derived 47832c strain (GenBank: KC618403) was used as a reference sequence to design suitable siRNAs. This HEV-3c subtype is adapted to cell culture, and an established persistently infected cell line could be used to mimic chronic infection. This strain was, therefore, selected for all further experiments in the current study. The siRNA design was done in silico using multiple online software tools (Horizon Discovery, Eurofins Genomics, OligoWalk [50], siDirect [51]) to take into account both the sequence and structure of the target RNA [52]. Regions with overlapping siRNAs were chosen as siRNA targets, and a total of 6 siRNAs were selected for further analysis. The Nucleotide BLAST program was utilized with default parameters to confirm the absence of seed sequences in the human transcriptome. The control siRNA, siCon, was verified to not align with any sequences found in the viral or human genome [53]. All siRNAs were purchased from Microsynth AG (Balgach, Switzerland), and their corresponding sequences are shown in Table 2.

The silencing efficacy of the siRNAs was assessed through reporter assays. Two distinct vectors were generated for each target, both expressing the firefly luciferase reporter. The HEV-3c-ORF3 and HEV-3c-Hel sequence, both derived from the patient-derived and cell culture-adapted virus sequence of the 47832c strain, were synthesized by Thermo Fischer Scientific (Invitrogen, Carlsbad, CA, USA) and sequences were inserted individually downstream of the Renilla luciferase gene of the psiCHECK™ 2 vector (Promega, Madison, WI, USA) plasmid using the XhoI/NotI restriction sites in the MCS.

### 2.2. Cell Culture

The A549 subclone A549/D3, which is highly susceptible to infection with HEV strain 47832c [54] and A549 cells that have been persistently infected with HEV strain 47832c (A549/pers-HEV) [55] cells were cultured in minimum essential medium (MEM, Biowest, Nuaillé, France) supplemented with 10% fetal calf serum (FCS, c.c.pro, Oberdorla, Germany) for A549/D3 and with 2% FCS for A549/pers-HEV, 2 mM GlutaMAX (Gibco, Life Technologies Corporation, Paisley, UK), 1% MEM non-essential amino acids (NEAA, Biowest), and 1% penicillin–streptomycin (Biowest). HeLa cells (ACC 57, DSMZ, Braunschweig, Germany) were maintained in Dulbecco’s modified Eagle’s medium (DMEM, Biowest) supplemented with 10% fetal calf serum, 2 mM GlutaMAX, and 1% NEAA. All cell lines were cultured in T25 or T75 flasks in a 5% CO_2_ incubator at 37 °C.

### 2.3. Dual Luciferase Reporter Assay

HeLa cells at 10^5^ cells per well were plated in 24-well plates. After 24 h, co-transfection was carried out by adding 500 ng of the reporter plasmid psiCheck2-HEV-3c-ORF3 or psiCheck2-HEV-3c-Hel along with 40 nM siRNA, using Lipofectamine™ 2000 (Thermo Fisher Scientific, Invitrogen) according to the manufacturer’s guidelines. The efficacy of siRNAs in silencing was evaluated at 48 h post-transfection by measuring relative luciferase activity using the Dual-Luciferase Reporter Assay System (Promega), according to the manufacturer’s instructions.

### 2.4. Synthesis of 5′-Triphosphorylated siRNA

The 5′-triphosphorylated sense strands of the siRNAs (3p-siRNAs) were synthesized through in vitro transcription employing the AmpliScribe™ T7-Flash™ Transcriptionskit (LGC Biosearch Technologies, Lucigen Corporation, Middelton, WI, USA). Single-stranded RNA antisense oligonucleotides were ordered from Microsynth AG. Template DNA oligonucleotides with a T7 RNA polymerase promoter sequence followed by the siRNA sense sequence (Table S2) were ordered from Thermo Fisher Scientific (Invitrogen) and hybridized by incubation at 95 °C for two minutes, followed by cooling to 25 °C at −1 °C/minute. The 3p-sense strand of the siRNA was produced by in vitro transcription at 37 °C for 16 h according to the manufacturer’s instructions. The DNA template was then digested with DNase I, and the resulting 3p-sense strand was purified using the Monarch RNA Cleanup (New England Biolabs, Ipswich, MA, USA). Its concentration was measured using the NanoDrop™ 2000 (Thermo Fisher Scientific, Invitrogen). The 3p-sense strand was then paired equimolar with the antisense strand. After denaturation at 75 °C for two minutes, the mixture was cooled to 25 °C at −1 °C/minute. Non-hybridized strands were digested with RNase A (Macherey-Nagel, Düren, Germany) to ensure only double-stranded 3p-siRNAs, followed by purification using the Monarch RNA Cleanup Kit. The final concentration was determined using NanoDrop. To verify that the 3p-siRNAs were double-stranded, urea polyacrylamide gel electrophoresis (PAGE) was performed. A 15% urea polyacrylamide gel was loaded with 1 μg of each sample and separated at 100 V for approximately two hours. Gels were stained with ethidium bromide for 20 min and visualized with the Molecular Imager Gel Doc (Bio-Rad Laboratories, Hercules, CA, USA) using the Image Lab Software 6.1 (Bio-Rad Laboratories).

### 2.5. Transfection of siRNA and 3p-siRNA

For transfection, A549/D3 cells were seeded at 2.5 × 10^5^ cells per well in 12-well plates and 0.75 × 10^5^ cells per well in 24-well plates, reaching 60–70% confluence after 24 h for transfection. A549/pers-HEV cells were seeded at 1.25 × 10^5^ cells per well the day before transfection. The siRNAs were transfected at a concentration of 50 nM using Lipofectamine™ RNAiMAX Transfection Reagent (Thermo Fisher Scientific, Invitrogen) in either 24-well or 12-well plates, according to the manufacturer’s protocol. Similarly, polyinosin-polycytidylacid (Poly(I:C) HMW, InvivoGen, Toulouse, France) was transfected at concentrations of 0.5 or 0.25 ng/mL using Lipofectamine™ 2000 (Thermo Fisher Scientific, Invitrogen) as a control for RIG-I activation. Cells were incubated at 37 °C for 24 or 48 h, as indicated.

### 2.6. HEV-3c Production Using A549/Pers-HEV Cells

HEV particles were harvested as previously described [56] with the modification that persistent A549/pers-HEV cells were used, and the HEV-3c strain 47832c was isolated. Briefly, cells were washed with PBS, trypsinized, and resuspended in 500 μL fresh 2% MEM medium. This was followed by three freeze–thaw cycles to lyse the cells and release intracellular virus particles. The cell components were centrifuged at 16,000× *g* for 15 min, and the supernatant containing virus particles was transferred to a fresh reaction tube and stored at −80 °C. Infectious titers were determined by virus titration, followed by immunofluorescence, as previously described [57]. In contrast, infection with intracellular viral particles was performed at 37°, 5% CO_2_ overnight (16 h).

### 2.7. siRNA Antiviral Replication Assay

For HEV inhibition assays, A549/D3 cells were transfected with each of the siRNAs as described previously 24 h before the virus challenge. Infection with intracellular virus particles (HEV-3c strain 47832c) was performed at a multiplicity of infection (MOI) of 1.0 for 16 h. The infection was stopped by changing the medium and washing the cells with PBS. Supernatants and intracellular viral RNA were harvested 48 h or 96 h post-inoculation. Viral RNA was extracted from cell culture supernatant with the QIAamp Viral RNA Mini Kit (Qiagen, Hilden, Germany) and from cells with the RNeasy Mini Kit (Qiagen) according to the manufacturer’s instructions.

### 2.8. Reverse Transcription Quantitative PCR of HEV Samples

Viral RNA was quantified via HEV-specific reverse transcription quantitative PCR (RT-qPCR), specifically amplifying a conserved 70-nucleotide (nt) region within the ORF2/ORF3 genes, as previously reported [58]. An established internal standard was amplified in parallel to calculate the copy number with the corresponding cycle threshold (CT) value [59]. RT-qPCR was conducted on a LightCycler^®^ 480 Instrument (Roche, Basel, Switzerland) or on a CFX Opus 96 Real-Time PCR System (Bio-Rad Laboratories) utilizing the LightCycler^®^ Multiplex RNA Virus Master Kit (Roche). The reaction mixture, totaling 20 μL, consisted of 1× qRT-PCR Reaction Mix, 0.2 μmol/L probe, 0.4 μmol/L of primers, 1× RT Enzyme Solution, and 5 μL of the sample. Cycling conditions were set as follows: 15 min at 50 °C, 2 min at 95 °C, followed by 45 cycles of [10 s at 95 °C, 30 s at 63 °C].

### 2.9. Immunoblotting

A549/D3 cells in 12-well plates were washed twice with cold PBS 48 h after transfection and lysed in 50 μL RIPA buffer (Thermo Scientific, Pierce, Rockford, IL, USA) supplemented with Halt™ phosphatase inhibitor cocktail (Thermo Scientific, Pierce™). Protein concentration was determined using the Pierce™ BCA protein assay (Thermo Scientific, Pierce™) according to the manufacturer’s instructions. Protein separation was performed by SDS-PAGE using Mini-PROTEAN TGX Stain-Free gels (Bio-Rad Laboratories). Protein samples were prepared for running on polyacrylamide gels by adding 4X LDS sample buffer and heating at 95 °C for five minutes. Subsequently, 60 μg of the samples and 3 μL of CozyHiTM Prestained Protein Ladder (highQu GmbH, Kraichtal, Germany) were loaded onto the gel. Proteins were transferred from the SDS gel to a PVDF membrane using the semi-dry blotting method at 70 mA for 75 min. Subsequently, blocking was performed for one hour at room temperature in 1% BSA blocking buffer. Incubation with primary antibodies against RIG-I (#3743-1:1000, Cell Signaling Technologies, Danvers, MA, USA) and actin (#A5441–1:15,000, Sigma-Aldrich, Saint Louis, MO, USA) diluted in blocking buffer was performed overnight at 4 °C. The membranes were then washed three times with TBS T and incubated with HRP-conjugated secondary antibodies anti-rabbit (#31460–1:10,000, Thermo Scientific, Pierce™) and anti-mouse (#31430–1:10,000, Thermo Scientific, Pierce™) for 1 h at room temperature. Chemiluminescence detection was performed using the Pierce ECL Western Blotting Substrate Kit (Thermo Scientific, Pierce™) according to the manufacturer’s instructions, and images were captured on the ChemiDoc™ MP Imaging System (Bio-Rad Laboratories) using Image Lab Software 6.1 (Bio-Rad Laboratories).

### 2.10. Statistical Analysis

All data are presented as mean and standard deviation (SD) of three independent experiments. Statistical significance was determined by Brown–Forsythe and Welch’s one-way analysis of variance (ANOVA) followed by Dunnett’s multiple comparisons using GraphPad Prism version 8.0.2 for Windows (GraphPad Software).

## 3. Results

### 3.1. Design of HEV-3c-ORF3 and HEV-3c-Helicase Sequence Targeting siRNAs

For the development of siRNAs against HEV, the most common genotype in Europe, HEV-3 was selected. In order to identify suitable regions for siRNA design, a sequence alignment of existing HEV genotype 3 subtypes was performed (Figure 1B). Only a few conserved regions within the HEV-3 genome were identified and, therefore, a large part of the genome is not suitable as a target sequence for siRNA due to the risk of rapidly emerging escape mutants. The regions of the helicase and ORF3 appear to be the most highly conserved and are presumably the least susceptible to escape mutations. Once the HEV target sequences (helicase and ORF3) were selected, several online programs (Eurofins Genomics, Horizon Discovery, OligoWalk Web Server, siDirect2.0) were used to identify functional siRNA for the HEV-3c strain 47832c. Each program uses specific bioinformatic selection criteria. Figure 1C summarizes the results of all online tools. For each target region, the three siRNA sequences with the highest overlap within the programs were selected (Table 2). The Nucleotide BLAST program was utilized with default parameters to confirm the absence of seed sequences in the human transcriptome. The control siRNA, siCon, was verified to not align with any sequences found in either the viral or human genome [53].

### 3.2. RNAi-Mediated Targeting of the ORF3 and Helicase of HEV in Reporter Assays

**Figure 2:**
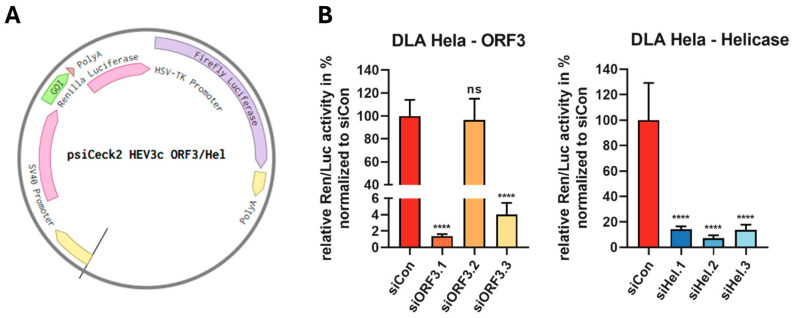
Relative change in luciferase activity by the designed siRNAs. A dual luciferase reporter assay was used to evaluate the silencing activity of the designed siRNAs. (**A**) Graphic representation of the psiCheck2 vectors where GOI is the HEV-3c ORF3 or HEV-3c helicase DNA sequence. (**B**) Co-transfection of dual-luciferase vectors (500 ng) and siRNA (50 nM) was performed in HeLa cells. The relative Renilla/Firefly (Ren/Luc) activity was determined 48 h after transfection. The relative activity was normalized against a non-regulatory control (siCon) and set to 100%, ns = not significant, **** *p* ≤ 0.0001.

The activity of the selected siRNAs was evaluated via a dual luciferase assay (DLA). The target sequence HEV-3c ORF3 or HEV-3c helicase was cloned into the psiCheck2 vector (Figure 2A) and co-transfected into HeLa cells with the corresponding siRNAs. The relative Renilla/Firefly activity was quantified 48 h after transfection, normalized against a non-regulatory control (siCon), and set to 100%. A significant reduction, each of approximately 90% in *Renilla* luciferase expression, was achieved with the three helicase-targeting siRNAs. Of the siRNAs targeting the ORF3, only two of the three were found to be active. The reduction in *Renilla* luciferase expression was ~98.6 ± 0.22% for siORF3.1 and ~96.0 ± 1.4% for siORF3.3. The two active ORF-specific siRNAs demonstrated a slightly greater efficacy than the helicase-specific siRNAs. In contrast, no reduction in *Renilla* luciferase expression was observed with siORF3.2 siRNA (Figure 2B). The results indicated that both the ORF3 and helicase sequences represent potential target sequences.

### 3.3. Inhibition of HEV-3c Replication Using ORF3- and Helicase-Targeting siRNAs in Persistently Infected A549/Pers-HEV Cells

**Figure 3:**
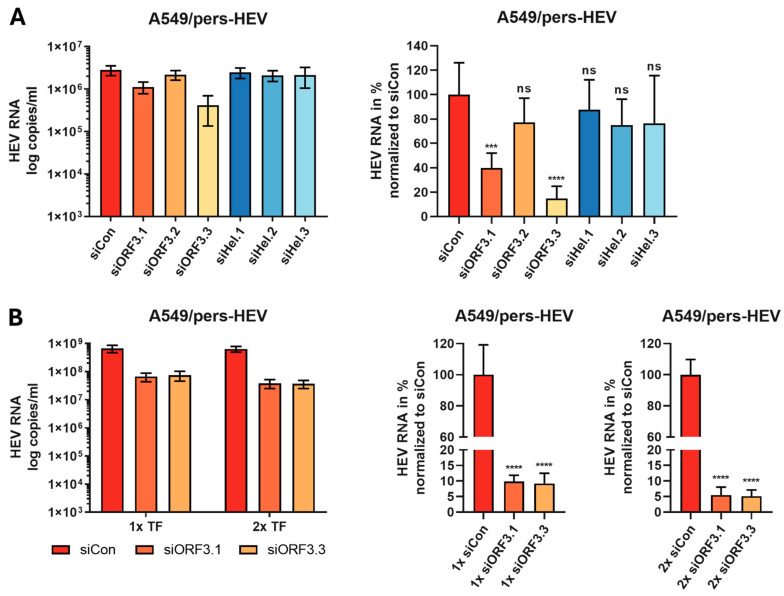
Changes in viral copy number by siRNA treatment of A549/pers-HEV cells. The viral RNA copy number was determined by qPCR after siRNA transfection of A549/pers-HEV cells. Transfection of 50 nM siRNA was performed 24 h after cell seeding. (**A**) At 48 h post-transfection, viral RNA was isolated, and the viral copies/mL were determined by qPCR. (**B**) A second transfection (TF) was then conducted 48 h after seeding. Viral RNA was isolated 96 h after the initial transfection, and the viral RNA was determined by RT-qPCR. The viral RNA was normalized against a non-regulatory control (siCon) and set to 100%. Mean ± SD of three independent experiments (*n* = 3) are shown. ns = not significant, *** *p* ≤ 0.001, **** *p* ≤ 0.0001.

Having shown that the siRNAs were active in reporter assays, we tested the siRNAs against infectious HEV. For this, the siRNAs were transfected once into A549/pers-HEV cells. These cells are persistently infected with the HEV-3c genotype isolated from a patient and adapted to the A549 cell line [55]. The viral load was determined 48 h after transfection by RT-qPCR. These experiments revealed some results that differed from those obtained with the dual luciferase assay (Figure 3A). Unexpectedly, no substantial reduction in viral load was observed for the siRNAs targeting the helicase sequence. In contrast, the siRNAs siORF3.1 and ORF3.3 directed against the ORF3 sequence showed a pronounced and significant reduction in viral copies down to 40% and 15% relative to the control, respectively.

Based on these results, the siRNAs ORF3.1 and ORF3.3 were used for further optimization experiments. The next step was to test whether a longer incubation period and multiple infections can improve silencing. To this end, A549/pers-HEV cells were transfected with siRNAs, and the viral RNA level was determined 96 h after the initial transfection. In additional experiments, cells were transfected with siRNA 48 h after the first transfection. The viral RNA was quantified by RT-qPCR. The second transfection 24 h after the first one resulted in a significantly improved reduction in the number of viral genomes. As can be seen in Figure 3B, the prolonged cultivation time of 96 h with a single transfection resulted in a further reduction of the viral load to approximately 9% for siORF3.1 and siORF3.3. A double transfection improved the inhibition to approximately 5% (Figure 3B).

### 3.4. Inhibition of HEV-3c Replication with Antiorf3-siRNAs Followed by HEV Infection

**Figure 4:**
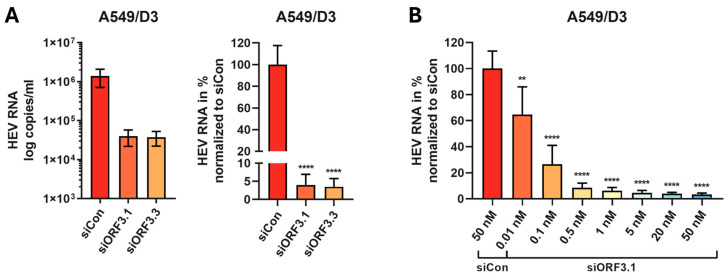
Inhibition of HEV replication by siRNA in infected A549/D3 cells. Cells were transfected and subsequently infected with viral isolates. At 96 h post-infection, viral RNA was isolated, and viral copies/mL were determined by qPCR. The viral copy number was normalized against a non-regulatory control (siCon) and set to 100%. (**A**) Transfection with 50 nM siRNA was performed 24 h after seeding. Infection was performed 24 h after transfection with viral isolates at an MOI of 1.0. (**B**) The A549/D3 cells were transfected with different concentrations (0.01 to 50 nM) of siORF3.1, and 24 h after transfection, the cells were infected with virus isolates at a MOI of 1.0 for 16 h. Mean ± SD of three independent experiments (*n* = 3) are shown. ** *p* ≤ 0.01, **** *p* ≤ 0.0001.

In addition to the A549/pers-HEV cell line, we also investigated whether the functional siRNAs could inhibit HEV replication in A549/D3 cells. A549/D3 cells were seeded and transfected with 50 nM siRNA after 24 h. After an additional 24 h, the A549/D3 cells were infected with HEV isolates from A549/pers-HEV cells at a MOI of 1.0. At 96 h post-infection, viral RNA was isolated, and viral load was determined by qPCR. Both siRNAs resulted in a significant inhibition of viral replication by approximately 96%. These experiments confirm the high potency of the designed siRNAs (Figure 4A).

Subsequently, the concentration-dependency of the inhibition of viral replication by siORF3.1 was performed. Different concentrations of the siRNA ranging from 0.01 to 50 nM were transfected. HEV infection was again performed at an MOI of 1 using HEV isolates from A549/pers-HEV cells. Figure 4B shows a clear concentration-dependent reduction of the HEV RNA level. Virus inhibition was still approximately 65% at a concentration as low as 0.01 nM, indicating a very high antiviral potency of siORF3.1.

### 3.5. RIG-I Activation and Inhibition of HEV Replication with 5′ Triphosphate siRNA

**Figure 5:**
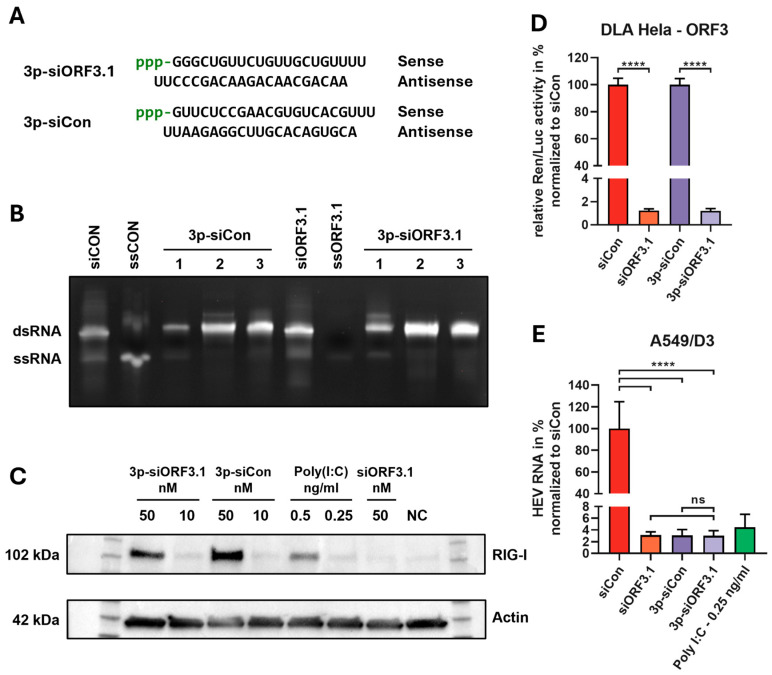
Design, synthesis, and functional testing of 5′ triphosphate siRNA. (**A**) Sequence and structure of 3p-siORF3.1 and 3p-siCon. The siRNA forms a 19 bp double strand with a UU overhang at the 3′-end and a triphosphate at the 5′-end of the sense strand. (**B**) Successful hybridization and purity of 3p-siRNAs (3p-siORF3.1 and 3p-siCon) were verified by a 15% urea polyacrylamide gel (1 = hybridization, 2 = RNase A digestion, 3 = final purification). The chemically synthesized siRNAs (siORF3.1 and siCon) and the antisense single strands ssORF3.1 and ssCon were used as controls. (**C**) Efficiency of RIG-I expression by Western blot analysis. A549/D3 cells were stimulated for 48 h with different concentrations of 3p-siCon, 3p-siORF3.1 (10 and 50 nM), poly I:C (0.5 and 0.25 ng/mL) or siORF3.1 (50 nM). Protein extracts from non-transfected A549/D3 cells served as negative control (NC). Actin was used as an internal control. (**D**) Dual-luciferase reporter assays were used to evaluate the silencing activity of the designed siRNAs. Co-transfection of dual-luciferase vectors (500 ng) and siRNA (50 nM) was performed in HeLa cells. The relative Renilla/Firefly (Ren/Luc) activity was determined 48 h after transfection. The relative activity was normalized against a non-regulatory control (siCon) and set to 100%. (**E**) Inhibition of HEV replication by 3p-siRNA: A549/D3 cells were transfected with the indicated siRNAs (siCon, siORF3.1, 3p-siCon, 3p-siORF3.1) at a concentration of 50 nM. The cells were infected with HEV at an MOI of 1.0, 24 h after transfection, and incubated for 16 h. At 96 h after infection, viral RNA was isolated, and viral load was determined by qPCR. The viral copy number was normalized against a non-regulatory control (siCon) and set to 100%. Mean ± SD of three independent experiments (*n* = 3) are shown. ns = not significant, **** *p* ≤ 0.0001.

HEV proteins have been shown to negatively affect the RIG-I signaling pathway [42,43,44]. A known RIG-I ligand, 5′-triphosphate RNA, was found to decrease HEV replication in infected A549 cells [45]. To exploit this effect, a strategy was developed in which tri-phosphorylated siRNA carries this modification only on the sense strand (Figure 5A), as it is removed from the antisense strand during RISC assembly by the Ago2 protein and is not required for silencing [60].

As the chemical synthesis of an siRNA strand with a 5′-3p is complex, the sense strand of the siRNA carrying the 5′-3p was generated by in vitro transcription and annealed to a chemically synthesized antisense strand. Using urea-PAGE, both 3p-siRNAs (3p-siORF3.1, 3p-siCon) were shown to be double-stranded after hybridization, RNase digestion, and purification (Figure 5B). To test the functionality of the 3p-siRNAs, the 3p-siRNAs (3p-siORF3.1 and 3p-siCon) and the purchased siRNAs (siORF3.1 and siCon) were transfected into A549/D3 cells. Poly I:C (0.5 and 0.25 ng/mL) was also transfected as a positive control, as it is known to activate RIG-I [61]. The cells were lysed 48 h after transfection and analyzed by PAGE and Western blot. Both 3p-siRNAs (3p-siORF3.1 and 3p-siCon) and poly I:C transfection resulted in strong expression of RIG-I in A549/D3 cells (Figure 5C). Furthermore, RIG-I activation appears to be concentration dependent, as the lowest concentration of 10 nM for both 3p-siRNAs did not result in any detectable activation of RIG-I. Poly I:C at 0.25 ng/mL also failed to induce RIG-I expression. Neither siORF3.1 (without 5′-triphosphate) nor untreated cells gave a positive band for RIG-I.

A DLA was performed (see Section 3.1) to verify that the newly generated 3p-siORF3.1 maintained its specific silencing activity against the HEV target site. Relative *Renilla*/Firefly activity was quantified 48 h after transfection, normalized against the respective control (siCon, 3p-siCon), and set to 100%. *Renilla* luciferase expression was significantly inhibited by both siORF3.1 and 3p-siORF3.1, with a significant knockdown of >98% (Figure 5C).

These results demonstrate that 3p-siORF3.1 is active against the target sequence in the dual-luciferase assay and that both 3p-siRNAs are effective stimulators of RIG-I in A549/D3 cells. To test the therapeutic efficacy of the modified siRNAs (3p-siORF3.1 3p-siCon) against HEV, A549/D3 cells were transfected with 50 nM of each of the siRNAs and subsequently infected with HEV as described in Section 3.4. The unmodified siORF3.1 and siCon, as well as 0.25 ng/mL poly I:C, served as controls. Viral RNA was isolated 96 h post-infection and quantified by RT-qPCR. Results were normalized relative to siCon. As expected, the modified siORF3.1 resulted in a significant inhibition of HEV replication to 3.1% (Figure 5D). Interestingly, compared to the control (siCon), 3p-siCon also resulted in a downregulation of HEV expression to 3% by stimulation of the RIG-I receptor alone. The inhibitory activity of 3p-siORF3.1 was comparable to that of siORF3.1 or 3p-siCon. The control transfection with poly I:C showed a similar inhibition of HEV replication to about 4.4%. These results demonstrate that HEV can be inhibited by specific RNAi-silencing or by unspecific induction of an immune response to a similar extent. While the combination of both mechanisms does not lead to a measurable increase in silencing activity, the double-edge strategy will still be of value to prevent the potential emergence of viral escape mutants induced by the siRNA.

## 4. Discussion

HEV infections have garnered increased attention recently. Although these infections are typically acute and self-limiting, they can progress to a chronic state with serious complications, especially in individuals with weakened immune systems. While there is no specific therapy available, ribavirin has been used off-label with some success [23,24]. Nevertheless, treatment with ribavirin frequently results in failure due to mutations in the viral genome [26,27,28]. Patients who do not respond to ribavirin therapy have no alternative treatments available, highlighting the need for new therapeutic options.

The cytosolic genomic RNA of RNA viruses, as well as the translated mRNA of DNA viruses, are excellent targets for therapeutic intervention using RNAi. Research groups have shown in various in vivo and in vitro studies that RNAi can successfully inhibit replication of virtually every medically relevant virus, including hepatitis B virus (HBV) [62], hepatitis C virus [63], coxsackievirus B3 [64], influenza A virus [65], adenovirus [66], human immunodeficiency virus [67,68,69], and the severe acute respiratory syndrome coronavirus type 2 [70,71].

In this study, we designed siRNAs against the ORF3 and the helicase domain of the HEV-3c strain. A comparison of HEV subtypes showed that the HEV-3 genome has very few conserved regions. In a comprehensive study, Ju et al. identified conserved regions across all genotypes of cis-acting elements, with 11 nucleotides in the 5′ coding region of ORF1 and six nucleotides in the 3′ coding region of ORF2 [72]. A very efficient siRNA targeting the ORF2 cis-element in HEV-1 was already found by Kumar et al. [33]. Since the siRNA target site requires a conserved region of at least 19 nucleotides, we opted to select a different region of the genome with a higher degree of conservation for designing our siRNAs. Only the ORF3 shows longer stretches of conservation (Figure 1). This is also reflected by the fact that mutations in ORF3 show reduced viral replication or eliminate HEV infectivity [18,73]. To ensure good siRNA efficiency, several online tools were used to obtain the best possible target sequence. A total of three siRNAs against ORF3 and three siRNAs against the helicase domain were selected. Using a dual luciferase reporter assay, we were able to show that all but one siRNA (siORF3.2) succeeded in significantly inhibiting luciferase activity (Figure 2). These experiments also demonstrated that the siRNAs against ORF3 showed better silencing than those against the helicase domain. All siRNAs were then tested against the genotype 3c strain 47832c adapted to cell culture. The A549/pers-HEV cell line persistently infected with the strain [55] was transfected with the siRNAs. Unexpectedly, the siRNAs against the helicase, found to be highly active in the reporter assays, did not function to inhibit the infectious virus to a satisfactory extent. The most likely explanation for this discrepancy is that the full RNA genome folds into a different structure than the isolated fragment that was used for the reporter assay, thereby preventing the siRNA targeting the helicase region from binding to their complementary sequences.

Further experiments with our selected siRNA demonstrate that prolonged cultivation and double transfection further improved the inhibition of viral replication. Studies by Bartlett et al. have shown that siRNA silencing lasts up to seven days in rapidly dividing cells and up to three weeks in non-dividing cells and that cell division is an important factor in RNAi [74]. This effect was further enhanced by Takahashi et al. using 2′-OMe-4′-thioribonucleoside modification to protect the siRNA from intracellular nucleases [75].

The present study focuses on the RNAi-mediated inhibition of the HEV-3c genotype. Previous studies have almost exclusively focused on HEV-4 and HEV-1, which are not particularly relevant in Europe [32,33,34,35]. Studies have also shown that subtype 3c is the most common genotype in Germany and the most relevant genotype for chronic HEV-infected patients [29,76,77,78,79]. Chronic HEV infections can be treated off-label with ribavirin, a guanosine nucleoside analog [23,24]. Despite some side effects, treatment is successful for many patients. However, ribavirin treatment leads to mutations and this may result in resistance to the treatment [26,27,28]. RNAi-based drugs may then serve as a second-line therapeutic for those who no longer respond to ribavirin treatment.

In our study, we used the A549 cell line for the infection experiments. This cell line is derived from adenocarcinomic human alveolar basal epithelial cells; it is widely used in HEV research [80,81,82,83,84]. Other studies have used hepatic cells [85,86]. However, various HCC-derived cell lines, including HepG2/C3A or Huh-7, are deficient in RIG-I signaling and were, therefore, not suitable for our study. We, therefore, decided to carry out the experiments with the widely used A549 cell line, but we will use HepaRG cell in further experiments, which have been shown to be infectable with HEV [87] and with which we even established a bioprinted 3D liver model [88].

For in vitro experiments to characterize antiviral siRNAs, it is a common procedure to transfect siRNAs first and infect the cells with the virus after a certain period to allow cellular uptake of the siRNAs [89,90]. While this procedure is highly artificial, as a therapeutic drug will be applied after the infection, it is necessary since viruses tend to lyse cells in vitro rapidly, preventing any attempt to simulate an ongoing infection in transfected cells. In the present study, we not only inhibited the virus with the pre-treatment strategy but also used the persistently HEV-infected cell line A549/pers-HEV [55] and achieved substantial and statistically significant inhibition of the ongoing infection.

In recent years, RNAi has become an increasingly important therapeutic approach, with numerous drug approvals every year [91]. The present study used Lipofectamine RNAiMAX for siRNA transfection into culture cells, an approach that cannot be used in patients. Efficient delivery of siRNAs to the target organ remains the major challenge in their therapeutic development. Delivery to the liver, however, is possible using existing techniques, in contrast to many other internal organs. Most of the siRNAs approved so far are covalently linked to N-acetylgalactosamine (GalNAc/NAc), which binds to asialoglycoprotein receptors on hepatocytes, facilitating specific delivery of siRNAs to the liver [30]. Furthermore, several RNAi therapeutics against chronic HBV infections are in various stages of development, which also use the GalNAc modification [92,93]. The siRNA presented here can thus be used as a GalNAc-linked molecule for future therapeutic application.

While RNAi is an efficient strategy to suppress viruses, it is well-known that the selection pressure of the siRNA treatment results in the rapid accumulation of escape mutations [94,95]. As a countermeasure, we chose to develop a combination strategy that uses the RNAi mechanism as a specific silencing pathway to inhibit the virus and, at the same time, activate the innate immune response by adding a triphosphate to the 5′ end of the siRNA. This functionalization is known to activate RIG-I and was shown to improve the anti-tumoral activity of an siRNA used to treat melanoma [96]. It has subsequently also been used for antiviral applications. Lin et al. found that a 3p-siRNA caused a significantly stronger inhibition of influenza A virus replication compared to an unmodified siRNA in A549 cells [46]. Other studies have shown that 3p-siRNAs against HBV achieved significantly higher inhibition of viral replication than siRNAs without 5′-triphosphate [48,62]. Animal studies also confirmed that 3p-siRNAs can inhibit the replication of influenza A viruses and HBV more effectively [46,48]. Research findings indicate that HEV proteins have a detrimental effect on the RIG-I signaling pathway [42,43,44], implying that HEV may manipulate the host antiviral response through this pathway.

We tested two different 3p-modified siRNAs and compared their activity to that of the unmodified counterparts: 3p-siORF3.1, which activates both specific RNAi against the HEV genome and RIG-I, and 3p-siCon, which induces only the RIG-I response without triggering RNAi. Initial DLA experiments confirmed that 3p-siORF3.1 retained full silencing activity when compared to siORF3.1, demonstrating its functionality despite the modification. RIG-I expression was analyzed by Western blot 48 h after transfection and showed that both 5′-3p-siRNAs can activate RIG-I.

The next step was to test the approach against the infectious virus. As RIG-I can also be activated by other synthetic ligands lacking a 5′-triphosphate, we used poly I:C, which is a positive control for RIG-I activation [97]. Treatment with poly I:C induced RIG-I as confirmed by Western blotting and efficiently inhibited HEV replication, highlighting the potential of RIG-I stimulation for virus inhibition. We then investigated the inhibitory activity of HEV-specific and control siRNA with and without 5′-triphosphate, respectively. While the unmodified control siRNA did not exert antiviral activity, the HEV-specific siRNA, as well as the 5′-triphosphate-modified control, efficiently inhibited the virus. The RIG-I activation by a modified siRNA alone is thus sufficient to suppress HEV. This finding is in line with an HEV-3a-replicon-based study by Xu et al., which has shown that 5′-triphosphate RNA can inhibit HEV RNA by approximately 67% and which also reported a significant reduction of HEV replication by overexpression of lentiviral RIG-I in HEV-infected Huh7.5, A549, and HepaRG cells [45]. Another study by Devhare et al. showed that transfection of the Huh7.5 cell line, which lacks active RIG-I signaling, with a RIG-I expression plasmid-reduced HEV-1 replication, while cells transfected with an empty vector showed higher HEV replication [98]. Our results support the hypothesis that 5′-3p-siRNA can effectively reduce HEV replication by activating the innate immune response after infection. The experiments of the current study were carried out with the 47832c strain, which is a clinical isolate that is a typical representative of HEV-3c, which is prevalent in Europe. As shown by Devhare et al. and Xu et al., other HEV strains are also susceptible to RIG-I signaling [45,98]. It will, therefore, be of interest to investigate in further experiments whether our strategy to inhibit HEV by 5′-3p-siRNAs will be as efficient in other strains as it is for HEV-3c.

A last resort therapy for RBV non-responsive patients is PegIFNα, but this therapy is contra-indicated for some organ transplant recipients, one of the highest risk groups for developing chronic HEV infections [21,22]. Our liver-specific targeted 3p-siRNA, which activates RIG-I and its respective Interferon I response, could fine-tune this therapeutic approach and provide an alternative to PegIFNα in organ transplant patients.

Interestingly, we did not observe an additive effect, i.e., improved virus inhibition, for the HEV-specific 5′-3p-modified siRNA compared to the unmodified active siRNA or the modified control. However, the modification was not primarily intended to improve the silencing activity but to combine two independent antiviral mechanisms. RNA viruses, with their poor replication fidelity, rapidly generate mutants capable of escaping repression by targeted siRNAs and thus become resistant to the treatment. Inhibition of viruses, particularly RNA viruses with a high rate of replicatory errors, will result in the enrichment of variants with mutations in the siRNA target site, which have become resistant to the treatment. This can be prevented by attacking the virus with a second, independent mechanism, the activation of the innate immune response by activating RIG-I signaling.

The siRNA inhibited the virus with high efficiency. While most in vitro studies developing RNAi antiviral approaches pre-treat cells with the siRNA and carry out the infection step afterwards, we were also able to inhibit HEV in a persistently infected cell line, which is closer to being a model for a chronic infection found in patients. Finally, we demonstrate that the addition of a triphosphate to the 5′ end of the sense strand of the siRNA also exerts high antiviral activity by activating RIG-I. We therefore suggest the use of a 5′-triphosphate modified siRNA for the treatment of HEV infections. The primary intention of using a double payload is not to increase antiviral activity but rather to prevent viral escape by combining two different antiviral mechanisms. As the liver is the best-suited organ for the delivery of siRNAs, this approach can develop into a rescue strategy for patients with chronic HEV infection that does not respond to the current standard treatments.

## Figures and Tables

**Table 1:** Summary of already published siRNA and shRNA against HEV.

Genotype	Organism	Target	Reference
4	Pig	RdRp	Huang et al. [32]
1	Human	Hel, RdRp, 3′CAE	Kumar et al. [33]
4	Human	ORF2	Huang et al. [34]
4	Pig	ORF3	Liu et al. [35]
3	Human	Met, Y, HVR, X, Hel, RdRp, ORF2	Zhang et al. [36]

**Table 2:** Sequences of designed siRNAs.

Name	Target	siRNA-Sequence
siORF3.1	ORF3	5′-GGGCUGUUCUGUUGCUGUUTT-3′ 3′-TTCCCGACAAGACAACGACAA-5′
siORF3.2	ORF3	5′-GGGUUGAUUCUCAGCCCUUTT-3′ 3′-TTCCCAACUAAGAGUCGGGAA-5′
siORF3.3 ^1^	ORF3	5′-CCUAUAUUCAUCCAACCAATT-3′ 3′-TTGGAUAUAAGUAGGUUGGUU-5′
siHel.1	Helicase	5′-GGAUGUUGAUGUGGUGGUUTT-3′ 3′-TTCCUACAACUACACCACCAA-5′
siHel.2	Helicase	5′-ACCGCAUUUGUUGCUACUATT-3′ 3′-TTUGGCGUAAACAACGAUGAU-5′
siHel.3	Helicase	5′-ACUUUCACGGAGACUACAATT-3′ 3′-TTUGAAAGUGCCUCUGAUGUU-5′
siCon	no target	5′-ACGUGACACGUUCGGAGAATT-3′ 3′-TTUGCACUGUGCAAGCCUCUU-5′

^1^ Sequence is identical to ORF3-siRNA2 from Lui et al. [35].

## Data Availability

Data are available upon reasonable request.

## Supplementary Materials

The following supporting information can be downloaded at: https://www.mdpi.com/article/10.3390/v16091378/s1, Table S1: Reference sequence for HEV-3 Subtype a-j; Table S2: DNA oligonucleotides for T7 in vitro transcription of 5’- triphosphorylateded siRNA.
