# Supplementary material for: Combining RNA Interference and RIG-I Activation to Inhibit Hepatitis E Virus Replication
Source: Viruses. 2024 Aug 29;16(9):1378. doi: 10.3390/v16091378 (PMC11435946; doi:10.3390/v16091378)
Supplement: Supplementary file 1 [file viruses-16-01378-s001.zip › viruses-3132666-supplementary.pdf]

Supplementary

Table S1. Reference sequence for HEV3 Subtype a–j

Subtype	Strain	GenBank
3a	Meng	AF082843
3b	JRA1	AP003430
3c	wbGER27	FJ705359
3e	swJ8-5	AB248521
3f	E116-YKH98C	AB369687
3g	Osh 205	AF455784
3h	TR19	JQ013794
3i	BB02	FJ998008
3j	Arkell	AY115488

Table S2. DNA oligonucleotides for T7 in-vitro transcription of 5′-triphosphorylateded siRNA.

Name	DNA Sequence (5′ → ‘3)
T7-ORF3.1 Sense	**TAATACGACTCACTATAG**GGCTGTTCTGTTGCTGTTTT<br>**T7-Promotor** I siRNA-Sense
T7-ORF3.1 Antisense	AAAACAGCAACAGAACAGCCCTATAGTGAGTCGTATTA
T7-siCon Sense	**TAATACGACTCACTATAG**TTCTCCGAACGTGTCACGTTT<br>**T7-Promotor** I siRNA-Sense
T7-siCon Antisense	AAACGTGACACGTTCGGAGAACTATAGTGAGTCGTATTA
